# Exploring the therapeutic potential of edible vegetables, fruits, and spices against cancer in various cell lines

**DOI:** 10.7150/jca.89539

**Published:** 2024-01-01

**Authors:** Md. Rahmat Ali, A.S.M. Ali Reza, Md. Anwarul Haque, Md. Jannatul Islam, Md. Rezwan Hossain, Md. Ibrahim Mollah, Md. Badrul Islam, Joy Sarker, Mamunur Rashid, Md. Golam Sadik, Donatella Cicia, Raffaele Capasso, Mohsin Kazi, AHM Khurshid Alam

**Affiliations:** 1 Department of Pharmacy, University of Rajshahi, Bangladesh.; 2 Department of Pharmacy, International Islamic University Chittagong, Bangladesh.; 3 Bangladesh Council of Scientific and Industrial Research (BCSIR), Rajshahi, Bangladesh.; 4 Department of Pharmacy, University of Naples Federico II, Naples, Italy.; 5 Department of Agricultural Sciences, University of Naples Federico II, Italy.; 6 Department of Pharmaceutics, College of Pharmacy, King Saud University, P.O. Box 2457; Riyadh 11451, Saudi Arabia.

**Keywords:** Anticancer activity, Apoptosis, Free radicals, Cancer, Vegetables, Fruits, Spices, Antioxidants

## Abstract

Cancer is rapidly becoming the leading cause of death globally. This study aimed to identify edible foods with cytotoxic and/or antioxidant activities that can prevent cancer when consumed in a regular diet. Sixty-eight edible foods were purchased from the local market, and the materials were extracted with 80% methanol. The cytotoxic activity of the extracts was evaluated using MTT on HeLa, H2228, HEK293, and H3122 cell lines. To study apoptosis, triple fluorescence labeling with DAPI, Annexin V, and propidium iodide was used. The phenolic content, antioxidant capacity, and free radical scavenging capabilities were studied using conventional spectrophotometric techniques. Among the edible foods, carrot, pointed gourd, wax gourd, ficus, apple, lemon, cumin seed, and white peppercorn showed moderate cytotoxicity in HeLa cells. The growth of HeLa cells was significantly inhibited dose-dependently by tomato, banana, Indian spinach, guava, lemon peel, and coriander (IC_50_, 24.54, 17.89, 13.18, 9.33, 1.23, and 2.96 µg/mL, respectively). Tomato, Indian spinach, lemon peel, and coriander exerted significant dose-dependent inhibition of H2228, HEK293, and H3122 cell proliferation. The tomato, Indian spinach, lemon peel, and coriander extracts induced HeLa cell apoptosis. White peppercorn, amaranth, apple, wax gourd, cumin seed, taro, and lemon peel contained significant amounts of polyphenols and showed high antioxidant activity. White peppercorn, apple, coriander, lemon peel, and ficus significantly scavenged DPPH free radicals (IC_50_ values of 10.23, 12.02, 13.49, 13.8, and 14.0 µg/mL, respectively). The overall results suggest that the daily intake of these antioxidant-rich cytotoxic foods can prevent or reduce the risk of cancer.

## 1. Introduction

Cancer is now the second-highest cause of mortality in developing nations and the top cause of death in newly industrialized countries 1. According to expected cancer statistics for 2023, 1,958,310 new cancer cases have been predicted in the United States 2. A study performed by the American Cancer Society reported that global cancer mortality is predicted to climb by 60% from 2020 to 2040, reaching 16.3 million deaths and 27.5 million new cases of the disease 3. One unresolved question is why cancer is quickly overtaking all other causes of mortality in wealthy nations such as the US and Canada. One reason may be the lack of early detection methods and improper use of medication, the evolution of drug-resistant cancers, or the fact that there are almost no biomarker molecules for the early prediction of cancer. Moreover, a growing body of data suggests that reactive oxygen species (ROS) have been implicated in a number of diseases, including cancer 4, 5. Under typical conditions, the formation of ROS and their scavenging by antioxidants are balanced, preserving the redox status of tissue homeostasis 6-8. Oxidative stress (OS), which is caused by an imbalance in the redox status, is characterized by an increase in the formation of free radicals such as H_2_O_2_ and OH. Free radicals have the potential to harm proteins, lipids, and even DNA within cells, leading to cancer 6.

Unfortunately, although some cancers are now curable, cancer and the difficulties associated with it may not be sufficiently treated by existing therapies. In the new era, treatments such as radiotherapy, surgery, immunotherapy, and targeted therapy have come to the forefront, some of which are highly selective 9. Moreover, traditional chemotherapy agents are nonselective, widely used, and highly toxic to cancerous cells and their surrounding normal cells, causing serious side effects and health-related complications 4, 10. Therefore, it appears urgent to find new alternatives for cancer therapy or prevention. There is a proverb that states, "Prevention is better than cure." How can cancer be prevented? Many factors increase cancer risks, including genetics, environmental factors, aging, and lifestyle choices such as smoking, physical inactivity, and diet. Notably, one-third of the overall cancer risk is attributed to lifestyle factors 11. According to another estimate, 30% of malignancies in Western nations are caused by dietary factors 12, making nutrition the second-most avoidable cause of cancer. Surprisingly, diet has been linked to up to 80% of certain malignancies, including large bowel, breast, and prostate cancers 12. Thus, extensive research has been performed to identify novel agents from natural sources, such as plants, fruits, vegetables, extracts, and biomolecules, for preventing and treating various cancers. Consuming the right kinds and amounts of edible foods with anticancer potential would be an excellent strategy for preventing cancer 13. Cancer risk can be significantly influenced by what we eat and do not eat. Certain dietary practices can significantly affect cancer risk, even though research frequently only finds connections between particular foods and cancer rather than proving cause-and-effect relationships. For instance, consuming a typical Mediterranean diet high in fruit, vegetables, and healthy fats such as olive oil helps reduce the risk of a number of common malignancies, including breast cancer. The risk of colon cancer is, however, increased by a diet that includes a plate of processed meat every day.

Diets based on foods of vegetable origin have been linked to improved health because they contain bioactive substances such as vitamins and flavonoids, which is supported by a substantial body of epidemiological and clinical research 14. Flavonoids and their metabolites not only directly inhibit ROS generation in the human body but also have direct free radical scavenging abilities 8, 15, 16. Fruits and spices used for culinary purposes also represent excellent sources of phytochemicals and natural antioxidant substances 17. Edible fruits and vegetables are also important for their inherent fiber, which has been proven to help reduce certain cancers. Moreover, numerous edible phytochemicals, such as sulforaphane and phenethyl isothiocyanate (cruciferous vegetables), resveratrol (grapes and grape products), and lycopene (tomatoes), have potential antioxidant and anticancer activities. Consequently, a variety of phytochemicals found in edible fruit, vegetables, and spices may exert integrated antioxidant processes that contribute to the health advantages of plant-based diets 17. Therefore, there is mounting interest in incorporating appropriate foods, food extracts, and phytochemical formulations from edible vegetables, fruits, spices, and plant sources into our regular meals to successfully manage and prevent OS and/or diet-related illnesses such as cancer 18, 19.

In this work, we examined the cytotoxic and antioxidant effects of 68 commonly consumed edible foods from the plant kingdom, including 30 vegetables, 17 fruits, and 21 spices, on various cancer cell lines and cell-free environments. Their cytotoxic activity was further confirmed by apoptosis analysis. Nine vegetables (sweet gourd, carrot, green banana, taro, amaranth, green taro, pointed gourd, wax gourd, and ficus), four fruits (apple, wood apple, banana, and lemon), and two spices (cumin seed and white peppercorn) showed moderate cytotoxicity on HeLa cells (cervical cancer). Interestingly, three vegetables (tomato, banana, and Indian spinach), two fruits (guava and lemon peel), and one spice (coriander) significantly reduced the growth of HeLa cells. The cytotoxic activity of these samples was confirmed by examining their activity in other cancer cell lines (HEK293 [human embryonic kidney 293], H3122 [human lung adenocarcinoma], and H2228 [human lung adenocarcinoma]). Interestingly, tomato, Indian spinach, lemon peel, and coriander significantly inhibited the growth of HEK293, H3122, and H2228 cells, and the anticancer activity was mediated by triggering the apoptosis of HeLa cells. Next, foods with minor to moderate anticancer potential were tested for their antioxidant and free radical-scavenging capacities. Surprisingly, white peppercorn, amaranth, apple, wax gourd, cumin seed, and lemon peel had significant amounts of polyphenols, although these foods, except lemon peel, had mild cytotoxic activity. Pointed gourd, coriander, wax gourd, tomato, and lemon peel had significant antioxidant activity, although these foods, except coriander and lemon peel, did not show remarkable cytotoxic activity. Moreover, white peppercorn, apple, wax gourd, lemon peel, ficus, and coriander showed significant scavenging action. In addition, lemon peel and coriander showed significant anticancer activity. The combined findings suggest that the identified foods with anticancer, antioxidant, and radical-scavenging actions could be regarded as potential sources of anticancer foods, and cancer prevention may be achieved by routinely consuming these edible foods.

## 2. Materials and Methods

### 2.1. Materials and reagents used in the different assays

The substances employed in this investigation were methanol, Folin-Ciocalteu reagent, sodium carbonate (Na_2_CO_3_), gallic acid (GA), 1,1-diphenyl-2-picrylhydrazyl (DPPH), butylated hydroxytoluene (BHT), sulfuric acid, sodium phosphate, catechin (CA), and ammonium molybdate, which were purchased from the local market in Bangladesh. Dulbecco's modified Eagle's medium (DMEM), fetal bovine serum (FBS), 3-(4,5-dimethylthiazol-2-yl)-2,5-diphenyltetrazolium bromide (MTT), phosphate-buffered saline (PBS), 4′,6-diamidino-2-phenylindole (DAPI), annexin V-fluorescein isothiocyanate (FITC), propidium iodide (PI), and dimethyl sulfoxide (DMSO) were purchased from Sigma‒Aldrich in India.

### 2.2. Collection of samples

The leaves, flowers, stems, and root bark of vegetables, fruits, and spices used daily were collected by purchasing them from the local market near the University of Rajshahi, Bangladesh.

### 2.3. Preparation of the samples

The gathered edible food items were properly cleaned with tap water to eliminate grime before being shed-dried for a number of days with brief periods of sun-drying. For easier grinding, these were subsequently dried for many hours at a very low temperature (32 °C). The dry components were ground into a coarse powder and stored at 25 °C for later use.

### 2.4. Extraction of the samples

The process described by Alam et al. 20 was used for the extraction to obtain bioactive chemicals contained in the edible foods. Before selecting the appropriate solvent, the dried coarse powdered materials were extracted with several solvents, such as ethanol, ethyl acetate, and chloroform. Based on the percentage yield, methanol was selected for extraction. Each coarsely powdered material was weighed out and put into an extraction bottle, which was then soaked with 80% methanol and run in an ultrasonicator bath (Analytical Lab Services, Chennai, India), which was automatically regulated for 15 min at ambient temperature. The process was repeated until the powder materials were colorless (usually 4-5 times). The resulting extracts were concentrated in a rotary evaporator (RE200, Bibby Sterlin Ltd., Stone, UK) at decreased pressure and 50 °C after being filtered with Whatman No. 1 filter papers.

### 2.5. Cell culture

Human HeLa, HEK293, H3122, and H2228 cells were purchased from the American Type Culture Collection (Manassas, VA, USA). The cells were routinely cultured in DMEM supplemented with 10% FBS in an atmosphere of 5% CO_2_ at 37 °C. In all experiments, exponential-growth cells were used.

### 2.6. Cell proliferation assay

The rate of cell growth was assessed using the MTT test. In 96-well plates, 5 × 10^3^ cells were grown for 48 h with samples containing 31.25-125 μg/mL. Each well received 20 μL of MTT (5 mg/mL diluted in PBS) to complete the treatment, which was then continued for an additional 4 h at 37 °C. The optical density (OD) was determined at 570 nm following the dissolution of the purple‒blue MTT formazan precipitate in 200 μL DMSO. The formula used to obtain the cell viability rate was as follows:

% Viable cells = OD of samples/OD of controls × 100

### 2.7. Detection of apoptosis

According to the procedure outlined by Miao et al. 21, DAPI, annexin V, and PI triple fluorescence labeling for cancer cell apoptosis was carried out. In short, HeLa cells were grown in media that contained 100 nM samples. The cells were rinsed twice in 0.01 M PBS and suspended in 200 μL binding buffer after 48 h. After that, the cells were incubated for 30 min at 4 °C in the dark with 10 μL each of DAPI, annexin V, and 5 μL of PI. An FV1000 confocal microscope was used to quickly detect the fluorescence of DAPI, annexin V, and PI (Olympus, Tokyo, Japan).

### 2.8. Estimation of the phenolic content

The total phenolic content was measured following the procedure outlined by Wolfe et al. 22, using Folin-Ciocalteu reagent as an oxidizing agent and GA as a standard. Each extract (2 mg) was dissolved in water to obtain a concentration of 2 mg/mL. Each sample and standard solution (500 µL each) were placed in test tubes. Then, 2.5 mL of Folin-Ciocalteu reagent and 2 mL of 7.5% weight (W)/volume (V) sodium carbonate (Na_2_CO_3_) were added. The reaction mixtures were vortexed and kept at RT for 25 min. The absorbance was read at 760 nm. The total phenolic content was determined by extrapolating a calibration curve that was constructed by preparing a GA solution. The estimation of the phenolic compounds was carried out in triplicate. The total phenolic content was expressed as mg GA equivalents (GAE) per g of dried sample.

### 2.9. Determination of antioxidant capacity

#### 2.9.1. Assessment of the overall antioxidant capability

The technique described by Prieto et al. 23, with a few minor adjustments, was used to ascertain the samples' overall antioxidant capacity. Each dried extract (2 mg) was dissolved in methanol to obtain a concentration of 2 mg/mL. Each sample and standard solution (500 µL) were put into test tubes and filled with the reaction mixture (3 mL), which contained 0.6 M H_2_SO_4_, 28 mM NaPO_3_, and 1% ammonium molybdate. The reaction mixture was then heated to ninety-five degrees Celsius for 10 minutes to finish the reaction. The absorbance at 695 nm is restrained by a spectrophotometer in comparison to a blank after cooling to RT (room temperature).

#### 2.9.2. DPPH radical scavenging assay

The free radical scavenging activities of the samples on the stable radical DPPH were estimated by the method described by Brand-Williams et al. 24. Briefly, 3.0 mL of a methanol solution of 0.1 mM DPPH was mixed with 2.0 mL of samples and a standard dissolved in methanol (100 μL) at concentrations ranging from 6.25-100 μg/mL. Samples and a standard solution (2 mL) were placed in the test tubes. After that, 3 mL of a 0.004% DPPH solution was added, and the samples were incubated for 30 minutes in the dark. At 517 nm, the absorbance was calculated in comparison to a blank solution. The DPPH free radical inhibition rate (%) was calculated as follows:

% DPPH free radicals = (1 - A_sample_/A_blank_) × 100

where A_blank_ represents the control reaction, which contained all reagents except the test material.

### 2.10. Statistical analysis

Each analysis was performed three times, and the data are presented as the mean ± SD. The significance between the test samples and the control was evaluated using Student's unpaired t test. Significant differences among different groups were evaluated using one-way analysis of variance (ANOVA), followed by Dunnett's post hoc test. *P* < 0.05 was deemed statistically significant. The statistical and graphical analyses were conducted using Microsoft Excel 2007 (Roselle, Illinois, USA) and R version 2.15.1 (http://www.r-project.org/). Experimental results were examined further for the Pearson correlation coefficient of phenolics with antioxidant and cell viability assays, and its significance was tested using Student's t test (*P* < 0.05).

## 3. Results

### 3.1. Determination of cytotoxic activity in cancer cell lines

**Figure 1:**
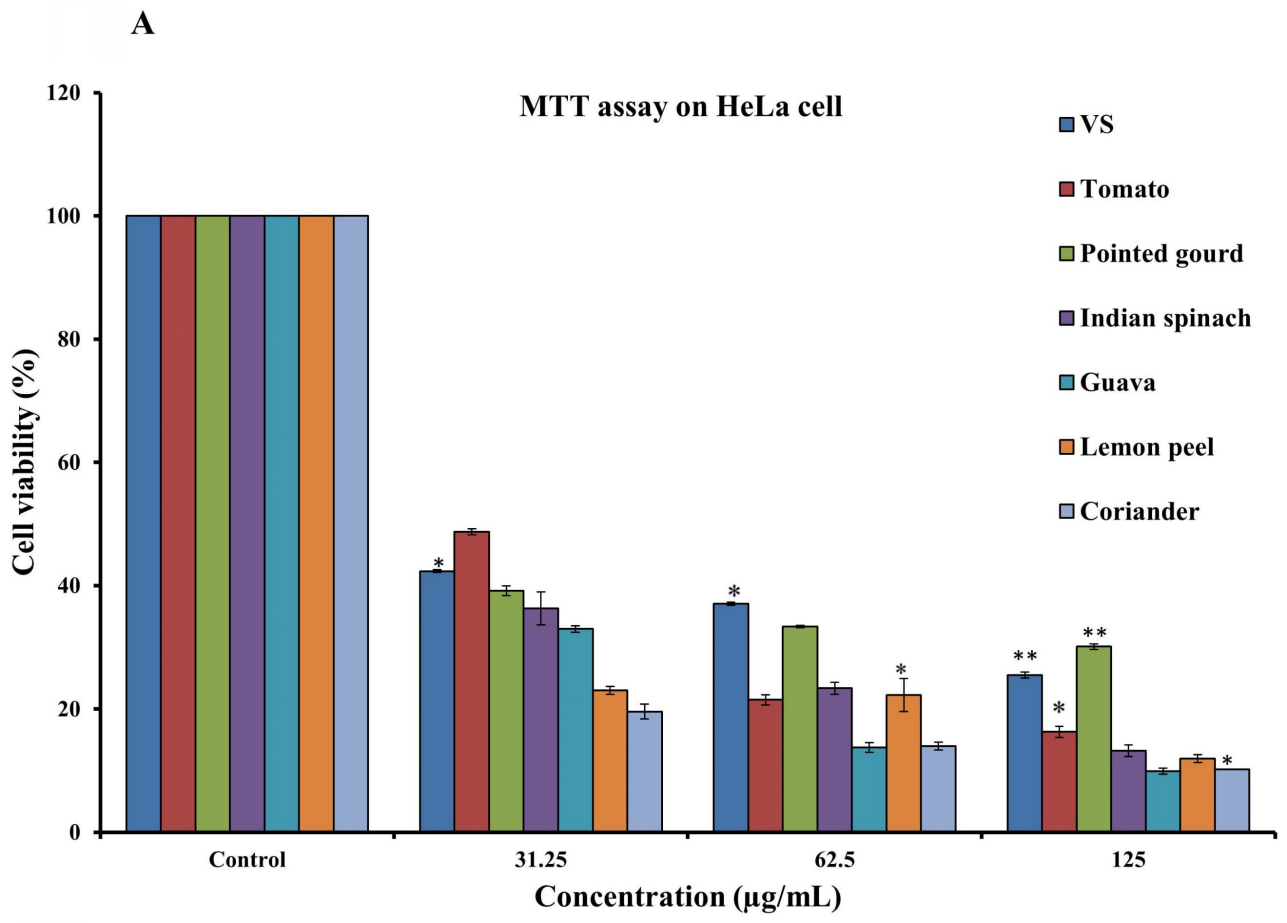

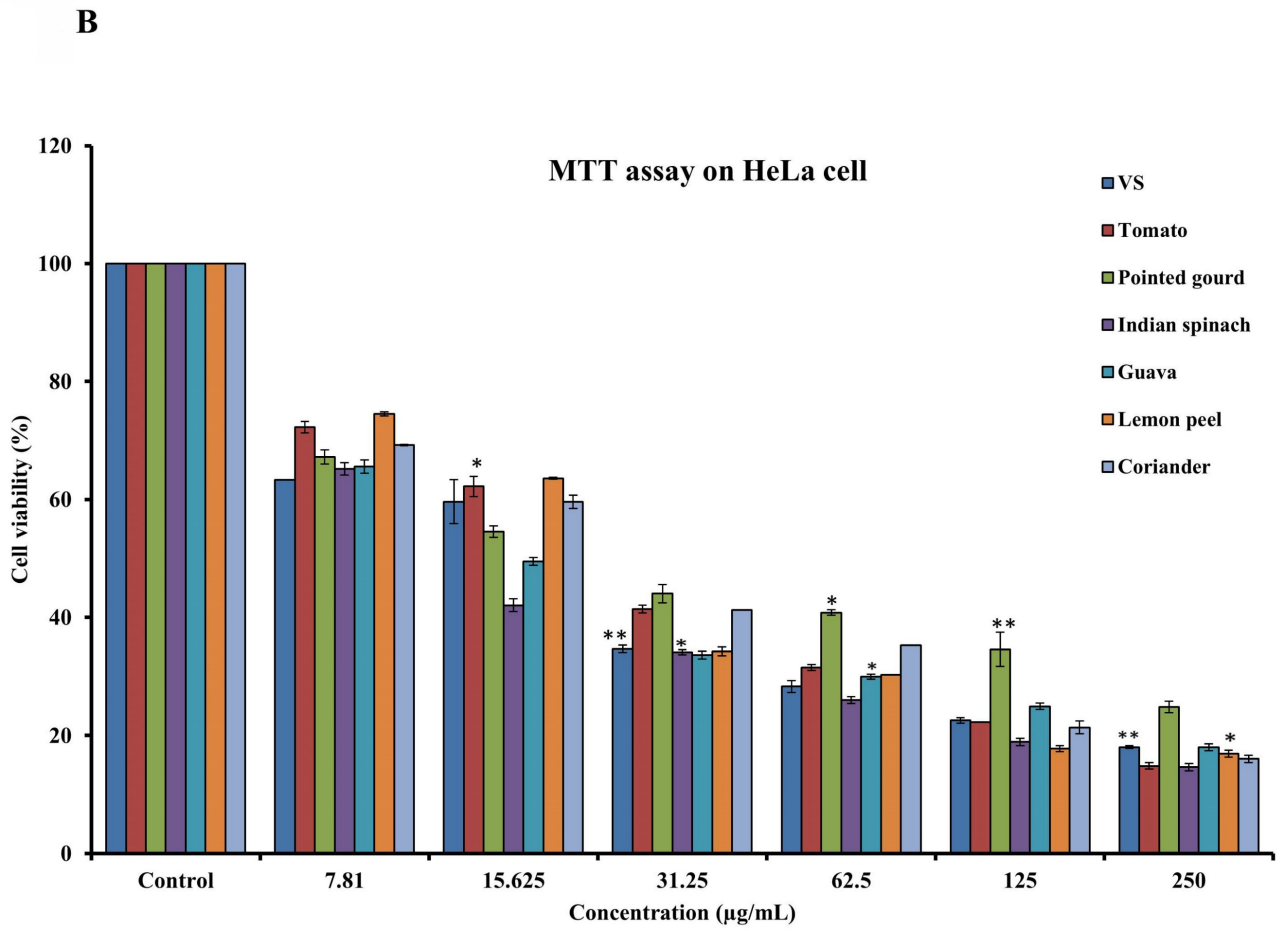
** A:** Determination of anticancer activity using the MTT assay on HeLa cells at concentrations of 31.25 to 125 µg/mL. For all dosages that were evaluated, data are presented as the mean ± SD (n = 3, *p < 0.05 and **p < 0.001). VS: Vincristine sulfate, tomato, pointed gourd, Indian spinach, guava, lemon peel, and coriander. **B:** Determination of anticancer activity using the MTT assay on HeLa cells at concentrations of 7.81 to 250 µg/mL. For all dosages that were evaluated, data are presented as the mean ± SD (n = 3, *p < 0.05 and **p < 0.001). VS: Vincristine sulfate, tomato, pointed gourd, Indian spinach, guava, lemon peel, and coriander.

**Figure 2:**
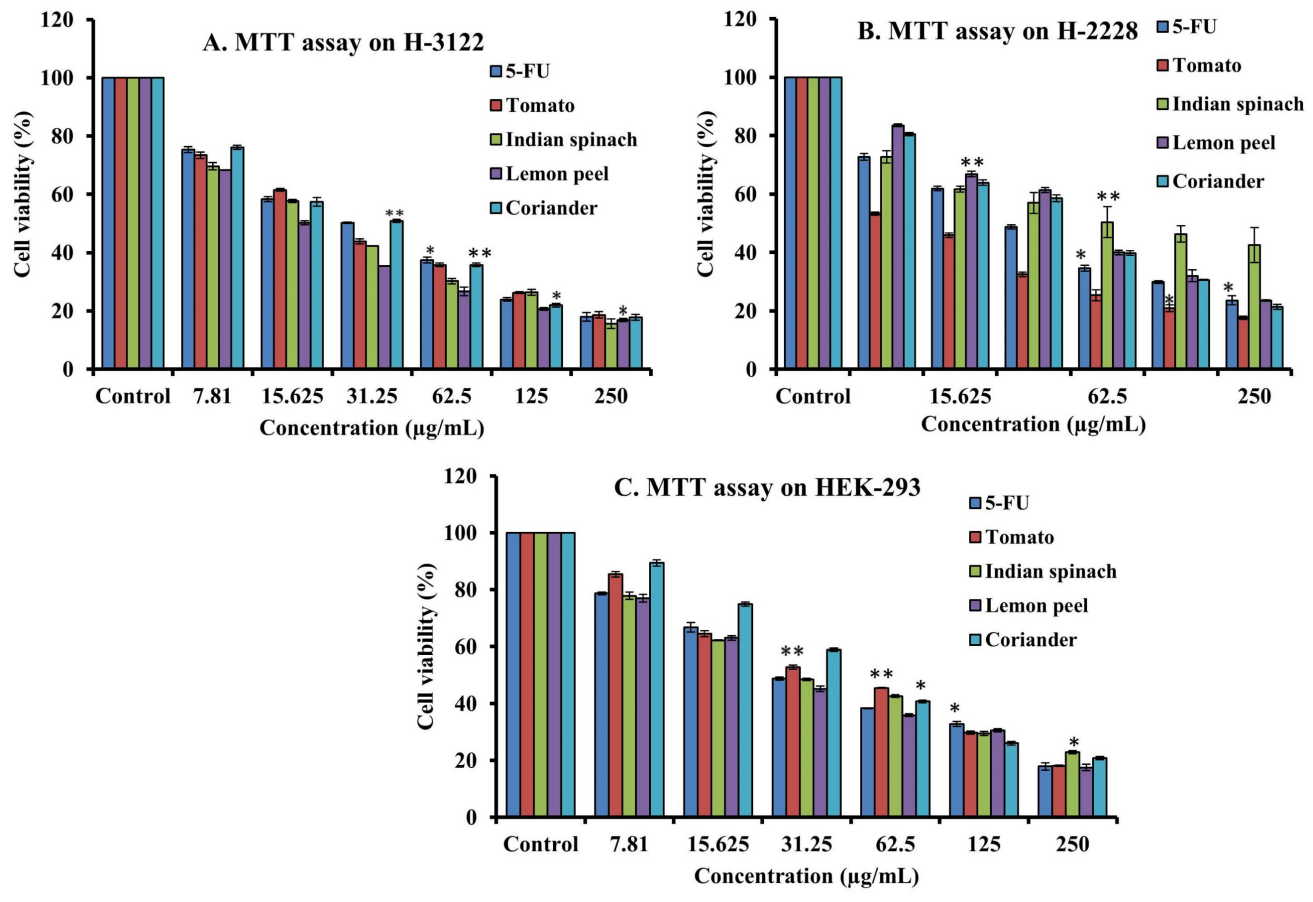
Determination of anticancer activity using the MTT assay on (A) H-3122, (B) H-2228 and (C) HEK-293 cells. For all dosages that were evaluated, data are presented as the mean ± SD (n = 3, *p < 0.05 and **p < 0.001). FU: 5-fluorouracil, VS: vincristine sulfate, H-3122: non-small cell lung cancer cell lines, H-2228: human lung carcinoma cell line and HEK-293: human embryonic kidney cell.

To assess the cytotoxicity of 68 dietary foods, i.e., 30 vegetables **(Supplementary Table S1)**, 17 fruits **(Supplementary Table S2)**, and 21 spices **(Supplementary Table S3),** on HeLa cells, the MTT assay was performed. Carrot, green banana, ficus, apple, lemon, and cumin seeds showed moderate cytotoxicity. Tomato, Indian spinach, guava, pointed gourd, coriander, and lemon peel showed strong dose-dependent inhibition of HeLa cell proliferation, with IC_50_ values of 24.54, 13.18, 9.33, 5.49, 2.96, and 1.23 µg/mL, respectively **(Figures 1A and 1B)**. The remaining samples had a mild or no effect on HeLa cell proliferation **(Supplementary Table S4)**. Next, we again used the MTT assay to determine whether tomato, pointed gourd, Indian spinach, guava, lemon peel, and coriander have cytotoxic activity on HEK293, H3122, and H2228 cells. Tomato, Indian spinach, lemon peel, and coriander exerted significant dose-dependent inhibition of H3122 **(Figure 2A)**, H2228 **(Figure 2B)**, and HEK293 **(Figure 2C)** cell proliferation, suggesting that the samples have differential functions against other types of cancer.

### 3.2. Measurement of apoptosis in HeLa cells

**Figure 3:**
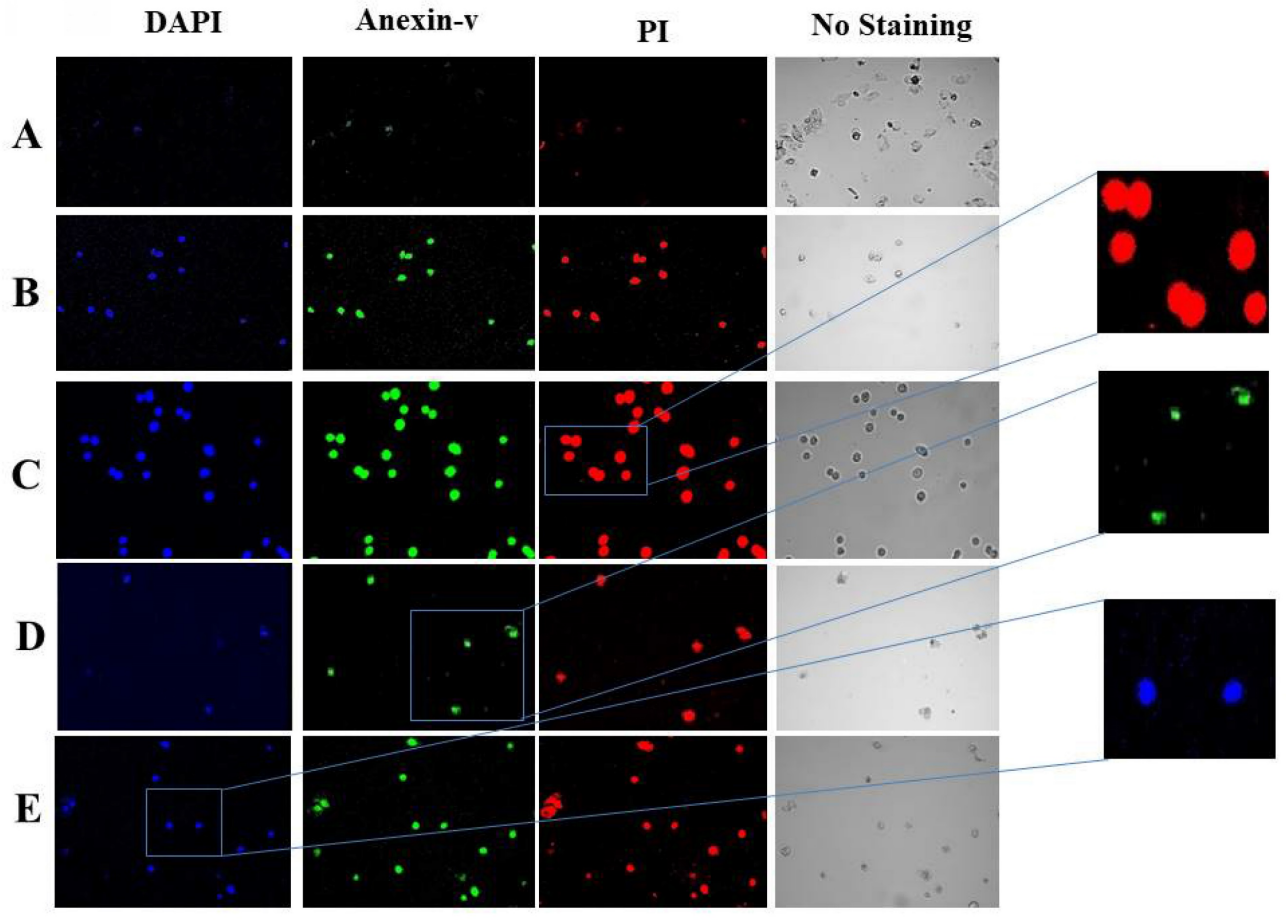
Representative images of DAPI, annexin-v, and PI triple florescence staining of apoptotic HeLa cells. The cell nucleus was visualized by a blue signal due to DAPI, annexin-v was visualized by a green signal, and PI was visualized by a red signal. (A) Control, (B) coriander, (C) lemon peel, (D) spinach, and (E) tomato.

Using DAPI, annexin V, and PI triple fluorescence labeling, we examined how tomato, Indian spinach, lemon peel, and coriander affected the apoptosis of HeLa cells. The ability of tomato, Indian spinach, lemon peel, and coriander to induce HeLa cell apoptosis was demonstrated by the lack of detectable annexin V and PI signals in control cells (without treatment), while treated cells had significant fluorescence densities **(Figure 3)**.

### 3.3. Estimating the amount of phenolics

The amounts of phenolics were calculated based on the GA standard curve (**Supplementary Figure S1**). We estimated the phenolic content of tomato, carrot, pointed gourd, green banana, Indian spinach, wax gourd, ficus, apple, guava, lemon juice, lemon peel, coriander, cumin seed, and white peppercorn, which showed moderate to strong cytotoxic activity. At a concentration of 100 µg/mL, the phenolic contents of wood apple, guava, green taro, carrot, tomato, lemon juice, Indian spinach, green banana, pointed gourd, coriander, ficus, lemon peel, taro, cumin seed, wax gourd, apple, amaranth, and white peppercorn were 1.43 ± 0.42, 2.90 ± 0.15, 3.28 ± 0.75, 3.90 ± 0.03, 4.15 ± 0.06, 4.89 ± 0.02, 6.02 ± 0.24, 7.11 ± 0.05, 7.52 ± 0.38, 8.27 ± 0.021, 9.60 ± 0.09, 11.16 ± 0.02, 11.23 ± 0.12, 11.64 ± 0.022, 15.35 ± 0.22, 19.00 ± 0.12, 22.23 ± 1.21, and 27.19 ± 0.02 mg GAE/g dried extract, respectively (**Supplementary Figure S2**).

Wax gourd, apple, amaranth, and white peppercorn had higher phenolic contents than the other samples; in contrast, wood apple, guava, green taro, carrot, tomato, and lemon juice showed the least cytotoxic activity. Hence, wax gourd, apple, amaranth, and white peppercorn might serve as good sources of antioxidants. The order of the amounts of phenolics was as follows: white peppercorn > amaranth > apple > wax gourd > cumin seed > taro > lemon peel > ficus > coriander > pointed gourd > green banana > Indian spinach > lemon > tomato > carrot > green taro > guava > wood apple.

### 3.4. Measurement of the overall antioxidant capability

**Figure 4:**
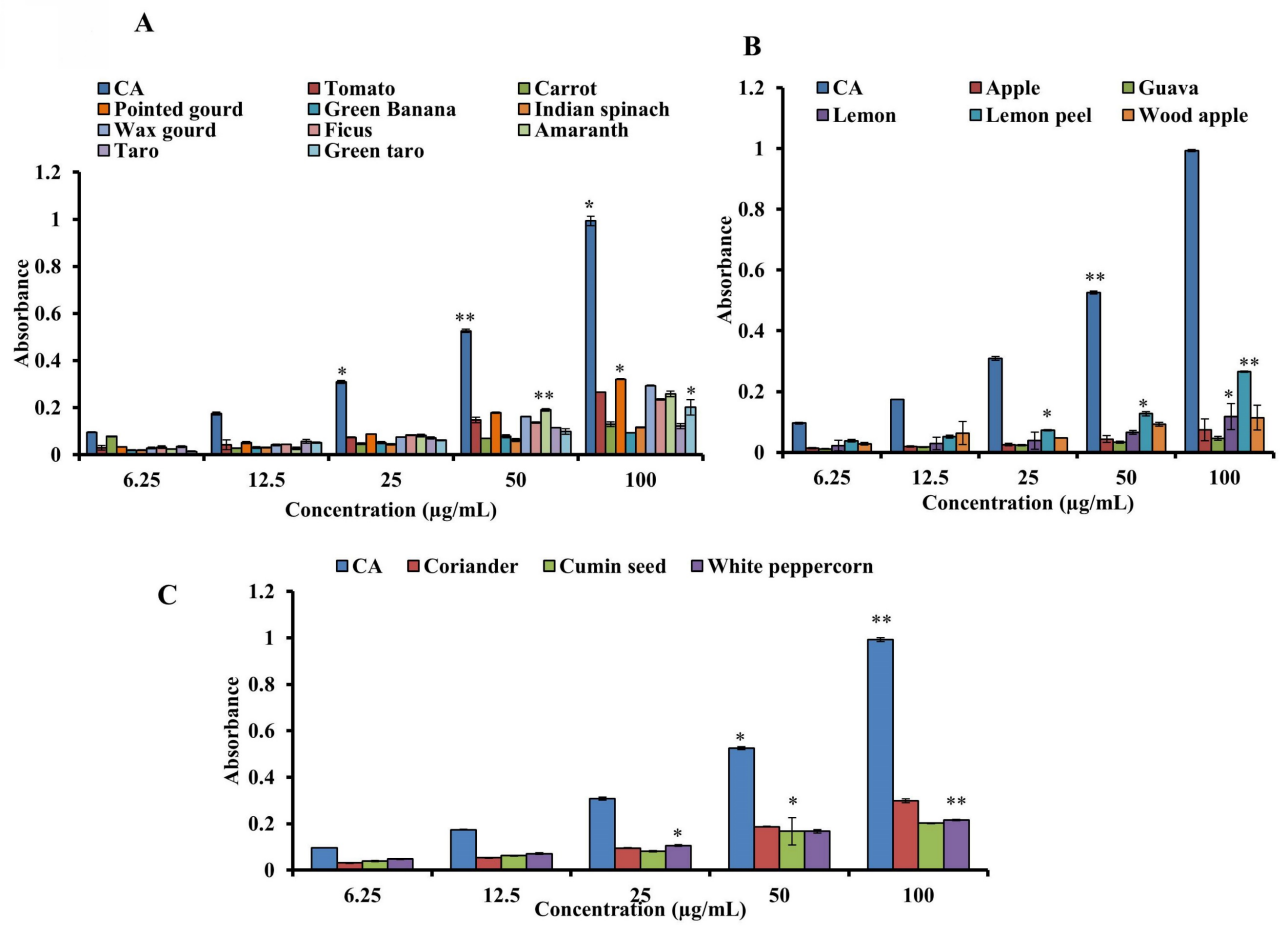
Determination of the total antioxidant capacity of standard CA and (A) vegetables, (B) fruits and (C) spices. For all dosages that were evaluated, data are presented as the mean ± SD (n = 3, *p < 0.05 and **p < 0.001).

The samples were assessed by the phosphomolybdenum method using CA as the standard to estimate antioxidant capability. **Figures 4A-C** show the standard's and samples' overall antioxidant capacities. At 100 µg/mL, pointed gourd had the highest activity (absorbance, 0.321 ± 0.002), followed by coriander (absorbance, 0.298 ± 0.008), wax gourd (absorbance, 0.293 ± 0.0021), tomato (absorbance, 0.266 ± 0.001), lemon peel (absorbance, 0.265 ± 0.002), amaranth (0.258 ± 0.012), ficus (absorbance, 0.235 ± 0.0021), white peppercorn (absorbance, 0.216 ± 0.003), cumin seed (absorbance, 0.202 ± 0.002), green taro (0.201 ± 0.033), carrot (absorbance, 0.129 ± 0.010), taro (0.121 ± 0.010), lemon juice (absorbance, 0.117 ± 0.012), Indian spinach (absorbance, 0.116 ± 0.002), wood apple (0.114 ± 0.041), green banana (absorbance, 0.093 ± 0.001), apple (absorbance, 0.074 ± 0.036), and guava (absorbance, 0.069 ± 0.020). The value of a standard was 0.993 ± 0.056 at 100 µg/mL. Our results demonstrate that all the samples have mild to moderate antioxidant activity. The antioxidant activity of the standards and the sample were in the following order: CA > pointed gourd > coriander > wax gourd >amaranth> tomato >lemon peel > ficus > white peppercorn > cumin seed >green taro > carrot >taro > lemon > Indian spinach > wood apple > green banana > apple > guava.

### 3.5. Determination of DPPH radical scavenging activity

**Figure 5:**
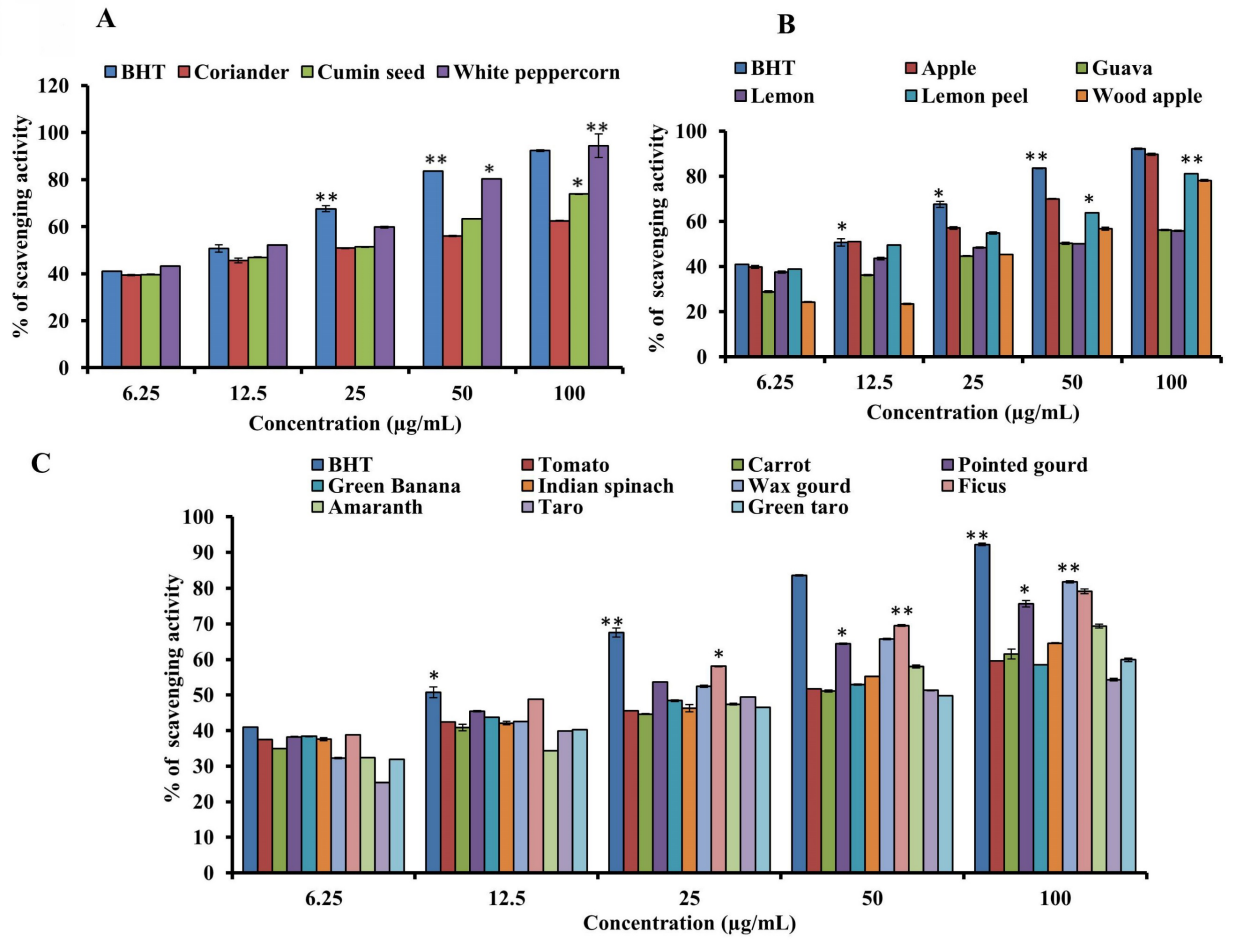
DPPH radical scavenging assay of standard BHT and (A) spices, (B) fruits and (C) vegetables. For all dosages that were evaluated, data are presented as the mean ± SD (n = 3, *p < 0.05 and **p < 0.001).

**Figures 5A-C** show the results of the activity to scavenge DPPH radicals of the samples and the standard BHT. The standard BHT (IC_50_ of 12.0 µg/mL) was higher than the white peppercorn (IC_50_ of 10.23 µg/mL), which had the maximum scavenging activity (93.73%). Lemon juice, guava, green banana, and tomato had the lowest scavenging activity. The IC_50_ values of guava, taro, lemon, green taro, carrot, wax gourd, tomato, green banana, wood apple, Indian spinach, amaranth, pointed gourd, cumin seed, ficus, lemon peel, coriander, apple, white peppercorn, and the standard were 46.77, 41.43, 37.15, 36.67, 33.88, 33.11, 33.11, 29.51, 29.46, 25.70, 17.23, 15.84, 15.85, 14.0, 13.8, 13.49, 12.02, 10.23, and 12.0 µg/mL, respectively. A lower IC_50_ indicates higher scavenging activity, suggesting that white peppercorn, apple, coriander, lemon peel, ficus, and cumin seed have significant DPPH radical scavenging activity.

### 3.6. Relationships and regressions among the amount of phenolics, antioxidants and cell survival potential

An excellent positive link among phenolic content, activity to scavenge DPPH radicals, and cell survival was found **(Supplemental Figures S3-S5).** Table 1 shows the relationships between phenolic content, antioxidants, and cell viability *(p value* < 0.001 and 0.05*)*.

## 4. Discussion

Despite improvements in modern medicine, cancer continues to be one of the leading causes of mortality globally. Modern science has not only enabled medical support to flourish to control or recover from leading fatal diseases, including cancer, but it has also reshaped our routine lifestyles, which directly or indirectly lead to various diseases by changing our food habits. A growing body of research indicates that lifestyle variables, specifically foods, exposure to chemical carcinogens (such as smoking), and physical inactivity, play a significant role in the onset of cancer 25. Moreover, the industrialized Western diet contains a range of mutagens and carcinogens that may operate by producing oxygen radicals and initiating cancer as well as other degenerative disorders 26. In addition, the consumption of high sugar and saturated fats induces OS by generating frequent ROS, which cause cancer, obesity, diabetes, and cardiovascular diseases 7. OS plays a critical role in controlling a variety of cellular activities, including cell division, proliferation, and death. It is evident that natural bioactive compounds derived from medicinal plants, vegetables, fruits, and spices can suppress ROS and act as promising anticancer agents due to their biocompatibility, biodegradability, lower toxicity, and reduced adverse effects. According to epidemiological studies, a high-fiber, low-fat diet that includes considerable amounts of fruits and vegetables lowers the risk of developing cancer 27. Hence, natural dietary ingredients might represent cutting-edge and intriguing cancer prevention or treatment alternatives. Approximately 23% of cancer cases can be prevented by increasing the dietary intake of fruits and vegetables 28. Fruits and vegetables contain micronutrients and other bioactive compounds, including carotenoids, folate, vitamins C, D, and E, vitamin B6, flavonoids, dietary fiber, and selenium, that potentially protect against cancer 29. These phytoconstituents may be able to inhibit the growth of cancer cells due to their propensity to trap free radicals, modulate detoxification enzymes, inhibit mutagenesis and proliferative processes, stimulate the immune system, and alter hormone concentration and metabolism 30. Because cancer is generally preventable, an early stage of cancer development is believed to have a greater chance of being prevented by consuming a nutritious diet, maintaining a healthy body weight, and exercising frequently 31. Hence, dietary nutrients that can alter bodily processes and stop civilization's lifestyle diseases have drawn increased interest from cancer experts.

Over two thousand years ago, in the 5^th^ century, Hippocrates said, “Let food be thy medicine”, and in the 1980s, a Japanese scholarly group proposed the idea of dietary foods as functional foods 32, 33. Hippocrates and the Japanese scholarly group both understood how crucial it is to have a healthy lifestyle that includes good food principles. Currently, including Japan, there has been a rapid increase in consumer interest in edible foods throughout the globe. The awareness of the benefits of dietary food is growing, and its demand is increasing in many countries. Several factors, such as increasing health concerns and growing health awareness about the value of edible foods, are propelling the demand for dietary food in Bangladesh and across the world 34, 35. These dietary recommendations encourage increasing the intake of food that contains phytochemicals, which have a significant impact on reducing chronic diseases, including cancer 36. In the current study, 68 edible foods were tested for anticancer activity in cervical, kidney, and lung cancer cells, which are the most prominent cancers not only in Southeast Asia but also worldwide. One of the most important findings of this study was that tomato, pointed gourd, Indian spinach, guava, lemon peel, and coriander exerted strong, dose-dependent inhibition of cervical cancer cell proliferation.

Moreover, tomato, pointed gourd, and Indian spinach showed potential anticancer activity in kidney and lung cancer cells. Michaud et al. reported that tomato-rich diets account for a reduction in the risk of several types of cancer, as tomatoes are a good source of secondary bioactive metabolites, including polyphenols, hydroxycinnamic acids, carotenoids, and vitamins 37. Our results reveal that tomatoes are a good source of antioxidants. Previous findings have suggested that lycopene and the other polyphenols present in tomatoes are mainly responsible for their antioxidant activity. Therefore, regular intake of tomatoes can prevent cancer by protecting lymphocyte DNA and serum proteins against oxidative damage 38. Another vegetable, the pointed gourd, is an important summer cucurbit vegetable in Bangladesh. It has abundant protein and vitamin A, and the fruits and seeds have strong potential for controlling some cancer-like conditions and hemagglutinating activity 39. In cervical cancer cell proliferation, pointed gourd showed potent anticancer activity. Moreover, it demonstrated the highest antioxidant capacity among all tested vegetables, fruits, and spices. Total antioxidant capacity, a measure of dietary antioxidant potential, is the number of oxidants that can be neutralized by a single molecule or food extract 40. Antioxidants may have a preventive impact against cancer because they are expected to lessen oxidative DNA damage and the ensuing genetic alterations 6, 8, 41. Here, we discovered a positive correlation between antioxidant activity and the total phenolic content of tomatoes and pointed gourds.

Supporting our findings, recent experimental evidence suggests that dietary antioxidants with redox potential synergistically interact against OS to control various degenerative diseases, such as cancer 42. Indian spinach is a leafy vegetable with high fiber, ash, calcium, vitamin, thiamine, riboflavin, phenolic, flavonoid, and niacin contents that is cultivated worldwide 43. Various in vitro and in vivo studies suggest that Indian spinach could be a good alternative for treating cancer, inflammation, atherosclerosis, stroke, heart disease, diabetes mellitus, multiple sclerosis, Parkinson's disease, and Alzheimer's disease 8. Hence, these experimental studies substantiate our findings on Indian spinach as a potent antioxidant and anticancer vegetable. Moreover, carrot, green banana, wax gourd, and ficus demonstrated reasonable to great anticancer and antioxidant activities. Previous epidemiological and clinical research on several fruits and vegetables supports our findings 27.

Fruits, such as vegetables, have a high polyphenol content and impressive antioxidant activity that may help lower the risk of cancer 44. In fact, numerous fruits and the main bioactive components of such fruits have the ability to fight cancer both in vitro and in vivo. According to Fu et al*.*, fruits contain a variety of polyphenolic compounds, including quercetin, kaempferol, phenolic acids, GA, chlorogenic acid, luteolin, ellagic acid, and protocatechuic acid 45, which can have a number of different biological impacts, including antioxidant, anti-inflammatory, antiviral, and anticarcinogenic actions 46. One of the most striking findings of this study was that apples contained the maximum phenolic contents along with potential antioxidant capacity. Recently, researchers have shown that polyphenols are the principal phytochemicals with anticancer properties in higher plants 47, 48. Diverse polyphenolic compounds may serve as cancer-suppressing and cancer-blocking agents by limiting the beginning of carcinogenesis and obstructing the growth and advancement of cancer 44. Guava and lemon peel dose-dependently inhibited the growth of cervical cancer cells. Interestingly, lemon peel also exerted significant dose-dependent inhibition of lung and kidney cancer cell proliferation, suggesting that fruits have diverse capacities for suppressing different types of cancer. Hence, guava and lemon peel could be considered in different approaches for preventing and treating various types of cancer, and the available seasonal fruits might be an important dietary source for preventing cancer.

Due to their flavor, taste, and color, spices have been frequently utilized as additives for thousands of years. Local folk practitioners use spices directly as medicine for a range of diseases. Srinivasan reported in a study that antioxidants derived from spices, such as curcumin from turmeric and eugenol from clove, control cellular OS, block ROS production, and disrupt signaling pathways 49. In the present study, coriander exerted strong dose-dependent inhibition of cervical cancer cell proliferation. Additionally, it markedly reduced the growth of kidney and lung cancer cells. Some species may reduce the incidence of some cancers by preventing the genesis, growth, and metastasis of cancer, according to epidemiological and investigative investigations 50. Therefore, spices could be a favorable source for new natural chemo drugs that can be widely used to manage adverse effects such as indigestion, nausea, vomiting, and a metallic taste caused by current chemotherapeutics 51. Hence, regular intake of spices, specifically coriander, in the daily diet can prevent cancer as well as increase the aesthetic appeal of edible food.

Additionally, DAPI, annexin V, and PI staining were used to assess the impact of tomato, Indian spinach, lemon peel, and coriander on cervical cancer cell apoptosis. In extremely late apoptotic cells, DAPI is employed as a marker of cell membrane permeability; annexin V labeling can indicate apoptosis at an earlier stage 21. In the current investigation, DAPI, annexin V, and PI signals were hardly visible in the untreated control cells, whereas they were strongly expressed in the treated cells, showing that the tested samples have the ability to cause cervical cancer cell apoptosis in both the early and late phases.

Overall, the phenolic content of dietary fruits, vegetables, and spices demonstrated a substantial and strong positive connection with antioxidants (DPPH) and cell viability assays *(p value* < 0.001 and 0.05*)*. We detected a high correlation between total phenolic content and DPPH radical scavenging, which is in line with the findings of Islam et al. 41. Our research clearly shows that phenolics are crucial parts of the foods we consume, and their abundance may be the reason for their beneficial pharmacological effects, such as anticancer activity.

People worldwide want to keep their bodies free from any diseases through a well-balanced lifestyle, including the proper intake of edible foods, but not through the use of medicines. Cancer, next to cardiovascular disease, is currently the second leading cause of death globally and will be the leading cause of death worldwide by 2030. The most unresolved question is why cancer is rapidly overtaking all other causes of death. The reasons may be as follows: 1) the lack of early detection methods; 2) nearly no biomarker molecules for early prediction; 3) the development of drug-resistant cancer; 4) the improper use of medicines; and 5) the toxicity of existing therapies. Therefore, there is an urgent need to find a new strategy that can tackle this issue. The selection of proper edible foods with anticancer activity in daily meals could be an alternative to preventing or reducing the risks of developing cancer. The most striking findings of this study were the inclusion of nearly all edible vegetables, fruits, and spices available on the market in Bangladesh in the study design, the investigation of their anticancer activity in multiple cancer cells, and the identification of a number of edible foods that showed anticancer potential. Although there is evidence that the identified edible foods have positive effects on a number of cancers, it may not be correct to state that the specific mechanisms and pathways causing these benefits are the same as those underlying the anticancer potentials of these edible foods. Therefore, more research is needed, including randomized controlled trials, to identify the phytochemicals in these foods that precisely show their modes of action and effectiveness in combating cancer. In addition, the availability of these natural foods in some areas may limit the capacity to extrapolate the findings of this study to the entire world and convert them to clinical applications. Nevertheless, the identified edible foods are thought to deserve further investigation because they contain a considerable amount of cytotoxic and antioxidant compounds.

## 5. Conclusion

We screened 68 dietary foods in several cancer cell lines using MTT. Tomato, pointed gourd, Indian spinach, guava, lemon peel, and coriander significantly suppressed the proliferation of cervical, kidney, and lung cancer cells. The data also show that the different samples have selective anticancer activity against different cancers. White peppercorn, apple, wax gourd, cumin seed, pointed gourd, tomato, and lemon peel contained significant amounts of polyphenols and showed high antioxidant activity. The overall phenolic content and anticancer properties of extracts were found to be significantly correlated with their antioxidant activity. Our study suggests that the identified vegetables, fruits, and spices should be utilized for preventing and treating different cancers in different ways. Moreover, our observations suggest a comprehensive preventive measure against different cancers through the utilization of edible vegetables, fruits, and spices. Foods obtained from plants are sources of many cancer-fighting compounds, such as fiber, vitamins, minerals, and phytonutrients. Rather than using supplements, incorporating vegetables, fruits, and spices into the daily diet could be a promising strategy for preventing or reducing the risk of developing cancer. Therefore, it is believed that the edible meals found definitely merit additional research to determine whether they can prevent cancer in rodent models.

## Supplementary Material

Supplementary figures and tables.

## Figures and Tables

**Table 1:** Correlation between the total phenolic contents and antioxidants as well as HeLa cell proliferation of dietary vegetables, fruits, and spices.

	Total Phenolic Contents (Correlation R^2^)	
Assays	DPPH	HeLa cell proliferation
Tomato	0.9981^**^	0.86976
Carrot	0.9563^**^	0.994
Pointed gourd	0.92505	0.89389^*^
Green Banana	0.92505^**^	0.84326
Indian spinach	0.96065^**^	0.96876
Wax gourd	0.93255^**^	0.85304
Ficus	0.87562^*^	0.764
Amaranth	0.91918^**^	0.9649
Taro	0.56858	0.99845^*^
Green taro	0.87617^*^	0.70192
Apple	0.96078^**^	0.71784
Guava	0.82035^*^	0.95005
Lemon	0.82113	0.72662
Lemon peel	0.94879^**^	0.96503
Wood apple	0.88379^*^	0.80212
Coriander	0.879^*^	0.84945
Cumin seed	0.93948^**^	0.89325
White peppercorn	0.93369^**^	0.92358

*Indicates significance at ^*^P<0.05, ^**^P<0.001. where DPPH assay =2,2-diphenyl-1-picrylhydrazyl assay
