# Supplementary material for: Exploring the therapeutic potential of edible vegetables, fruits, and spices against cancer in various cell lines
Source: J Cancer. 2024 Jan 1;15(3):577–89. doi: 10.7150/jca.89539 (PMC10777031; doi:10.7150/jca.89539)
Supplement: Supplementary file 1 — Supplementary figures and tables. [file jcav15p0577s1.pdf]

"Supplementary Material"

Research

Exploring the therapeutic potential of edible vegetables, fruits, and spices against cancer in various cell lines

Supplementary Table S1: List of vegetables

Sl. No.	Local Name	Scientific Name	English Name	Collected Part
1	Misti kumra	*Cucurbita moschata*	Sweet gourd	Flesh and Seeds
2	lau/kodu	*Lagenaria siccraria*	Bottle gourd	Peel
3	Begun	*Solanum melongena*	Brinjal	Whole brinjal
4	Dheros	*Abelmoschus esculentus*	Okra	Seed pods
5	Kakroll	*Momordica dioca*	Teasle gourd	Whole fruit
6	Korola	*Momordica charantia*	Bitter gourd	Fruits pod
7	Borboti	*Vigna sesquipedalis*	Cowpea	Edible green pods
8	Jhinga	*Luffa acutangular*	Ridge gourd	Unripe fruits
9	Shim	*Lablab niger*	Bean	Fresh fruits
10	Tomato	*Solanum lycopersicum*	Tomato	Fruits
11	Gajor	*Daucus carota*	Carrot	Young root
12	Potol	*Trichosanthes dioica*	Pointed gourd	Green fruits
13	Kach kola	*Musa paradisiaca*	Green Banana	Flesh of the fruit
14	Fulkopi	*Brassica oleracea var. botritis*	Cauliflower	White flesh, stems
15	Badhakopi	*Brassica olerace var. capitata*	Cabbage	Leaves
16	Palong shaak	*Spinacia oleracea*	Spinach	Leaves
17	Pui shaak	*Basella rubra*	Indian spinach	Whole plant
18	Lal shaak	*Amaranthus oleraceus*	Red amaranth	Whole plant
19	Shosha	*Cucumis sativus*	Cucumber	Fruits
20	Mula	*Rapsanus sativus*	Daikon	Roots
21	Alu	*Solanum teubersum*	Potato	Edible tuber
22	Chalkumra	*Benincasa hispida*	Wax gourd	Flesh of the fruits
23	Chichingga	*Trichosanthes cucumerina*	Snake gourd	Elongated flesh of the fruit
24	Mukhi kachu	*Colocasia antiquorum*	Taro	Edible corms and root
25	kolmi shak	*Ipomoea aquatica*	Water spinach	Whole plant
26	Danga	*Amaranthus tricolor*	Amaranth	Leaves and stems
27	Dumur	*Ficus hispida*	Ficus	Fruits
28	Kachushaak	*Colocasia esculenta*	Green Taro	Leaves
29	lau/kodu	*Lagenaria siccraria*	Bottle gourd	Flesh of the fruit
30	Mulashaak	*Rapsanus sativus*	Daikon	Leaves

Supplementary Table S2: List of Fruits

Sl. No.	Local Name	Scientific Name	English Name	Collected Part
1	Apple	*Malus pumila*	Apple	Flesh of the fruit
2	Peyara	*Psidium guajava*	Guava	Fruit
3	Malta	*Citrus sinensis L.*	Malta	Flesh of the fruit
4	Bell	*Aegle marmelos*	Indian Apple	Fruit
5	Kodbell	*Feronia limonia*	Wood Apple	Fruit
6	Jambura	*Citrus grandis*	Pomelo	Flesh of the fruit
7	Pepe	*Carica papaya*	Papaya	Flesh of fruit
8	Amloki	*Phyllanthus embelica*	Indian Goose Berry	Fresh fruit
9	Kamranga	*Averrhoa carambola*	Carambola	Fresh fruit
10	Kala	*Musa sapientum*	Banana	Soft flesh of theFruit
11	Amra	*Spondias dulcis*	Hog Plum	Flesh of the fruit
12	Dalim	*Puncia granatum*	Pomegranate	Fruit seeds
13	Boroi	*Zizyphus mauritiana*	Indian Jujube	Flesh of the fruit
14	Lebu	*Citrus limon*	Lemon	Juice of the fruit
14(a)	Lebu	*Citrus limon*	Lemon peel	Peel
16	Ata	*Anona squamosa*	Custard Apple	Flesh of the fruit
17	Kalojam	*Syzygium cumini*	Jambolan	Flesh of ripe fruit

Supplementary Table S3: List of Spices

Sl. No.	Local Name	Scientific Name	English Name	Collected Part
1	Ada	*Zingiber officinale*	Ginger	Rhizome ginger
2	Piyaj	*Allium cepa*	Onion	Whole onion
3	Rasun	*Allium sativum*	Garlic	Whole garlic
4	Halud	*Curcuma long*	Turmeric	Rhizomes
5	Marich	*Capsicum frutescens*	Chilli	Ripe fruit
6	Kalojira	*Nigella sativum*	Black cumin	The black seed
7	Dhoniy	*Coriandrum sativum*	Coriander	Whole plant
8	Jira	*Cuminum cyminum*	Cumin seed	Dried seed
9	Darochini	*Cinnamomum verum*	Cinnamon	Outer bark
10	Alachi	*Elettaria cardamomum*	Cardamom	Whole fruit
11	Lobongo	*Syzygium aromaticum*	Cloves	Dried aromatic flower buds
12	Joyfal	*Myristica fragrans*	Nutmeg	Whole fruit
13	Tejpata	*Cinnamomum tamala*	Bay leaf	Dried aromatic leaf
14	Mouri	*Pimpinella anisum*	Aniseed	Dried seed
15	Sada Sorisha	*Brassica juncea*	White masturd	Seed
16	Kalo Sorisha	*Brassica nigra*	Black mustard	Seed
17	Badam	*Arachis hypogaea*	Peanut	Seed
18	Sada Golmarich	*Piper nigrum*	White peppercorn	Whole white peppercorn
19	Kalo Golmarich	*Piper nigrum*	Black peppercorn	Whole black peppercorn
20	Kacha Marich	*Capscicum frutescens*	Green Chilli	Unripe fruit
21	Jayotri	*Myristica fragrans*	Mace	Dried aril

Supplementary Table S4: Determination of anticancer activity using the MTT assay of spices, vegetables and fruits on HeLa cells at concentrations of 31.25 to 125 µg/mL.

**Spices**				
				% of Viable Cells
	Concentration	0	Control	100
Std		31.25	VS	32.25701
		62.5		15.24771
		125		12.20401
		0	Control	100
1		31.25	Ginger	93.2975
		62.5		86.75623
		125		54.05507

		0	Control	100
2		31.25	Onion	86.9047
		62.5		64.12863
		125		56.83559
		0	Control	100
3		31.25	Garlic	78.76131
		62.5		61.23543
		125		56.3634
		0	Control	100
4		31.25	Turmeric	96.1587
		62.5		92.44041
		125		79.33898
		0	Control	100
5		31.25	Chilli	91.98001
		62.5		78.29599
		125		58.79626

		0	Control	100
6		31.25	Black cumin	94.01108
		62.5		77.63558
		125		59.88048
		0	Control	100
		31.25	Coriander	16.77878
7		62.5		11.22983
		125		8.655409
		0	Control	100
8		31.25	Cumin seed	51.63824
		62.5		34.79344
		125		24.66233
		0	Control	100
9		31.25	Cinnamon	91.43267
		62.5		81.24485
		125		67.57379
		0	Control	100
10		31.25	Cardamom	91.77225
		62.5		76.82887

		125		61.07056
		0	Control	100
11		31.25	Cloves	88.05088
		62.5		80.08286
		125		56.48114
		0	Control	100
12		31.25	Nutmeg	92.54429
		62.5		78.34062
		125		57.83801
		0	Control	100
13		31.25	Bay leaf	95.09276
		62.5		81.78231
		125		73.4922
		0	Control	100
14		31.25	Aniseed	84.44425
		62.5		77.46098
		125		65.1069
		0	Control	100
15		31.25	White masturd	99.41446
		62.5		73.93749
		125		21940.84

		0	Control	100
16		31.25	Black mustard	100.971
		62.5		67.58747
		125		59.82188
		0	Control	100
17		31.25	Peanut	93.39291
		62.5		81.34475
		125		62.88121
		0	Control	100
18		31.25	White peppercorn	58.2051
		62.5		37.32678
		125		22.09131
		0	Control	100
19		31.25	Black peppercorn	99.76218
		62.5		95.25383
		125		67.92528

		0	Control	100
20		31.25	Green Chilli	92.55737
		62.5		80.10269
		125		69.94824
		0	Control	100
21		31.25	Mace	99.87047
		62.5		81.99289
		125		69.46765
**Vegetables**				
		0	Control	100
Std		31.25	VS	19.4956
		62.5		16.13966
		125		12.92473
		0	Control	100
1		31.25	Sweet gourd	59.06682
		62.5		40.67777
		125		18.44306

		0	Control	100
2		31.25	Bottle gourd	99.3185
		62.5		91.93115
		125		71.32183
		0	Control	100
3		31.25	Brinjal	95.72074
		62.5		84.40625
		125		65.11443
		0	Control	100
4		31.25	Okra	99.82523
		62.5		80.64199
		125		62.18265
		0	Control	100
5		31.25	Teasle gourd	99.49948
		62.5		63.37708
		125		58.49078
		0	Control	100
6		31.25	Bitter gourd	92.07955
		62.5		79.55615

		125		68.62532
				0
		0	Control	100
7		31.25	Cowpea	99.39089
		62.5		82.84987
		125		70.39887
		0	Control	100
8		31.25	Ridge gourd	92.65866
		62.5		77.23968
		125		68.58913
		0	Control	100
9		31.25	Bean	98.52222
		62.5		78.18075
		125		61.53114
		0	Control	100
10		31.25	Tomato	38.1657
		62.5		21.30324
		125		12.49133
		0	Control	100
11		31.25	Carrot	59.33691

		62.5		47.72992
		125		30.47048
		0	Control	100
12		31.25	Pointed gourd	24.3162
		62.5		19.22233
		125		16.14668
		0	Control	100
13		31.25	Green Banana	64.69598
		62.5		40.60447
		125		29.82451
		0	Control	100
14		31.25	Cauliflower	99.13753
		62.5		79.8819
		125		67.21373
		0	Control	100
15		31.25	Cabbage	92.97222
		62.5		87.22539
		125		65.08051

		0	Control	100
16		31.25	Spinach	94.86792
		62.5		81.07047
		125		73.9925
		0	Control	100
17		31.25	Indian spinach	16.52513
		62.5		14.58845
		125		12.5664
		0	Control	100
18		31.25	Red amaranth	97.60953
		62.5		74.8806
		125		65.07533
		0	Control	100
19		31.25	Cucumber	108.6133
		62.5		90.306
		125		71.41232
		0	Control	100
20		31.25	Daikon	97.18301
		62.5		74.16312

		125		61.20539
		0	Control	100
21		31.25	Potato	99.87228
		62.5		92.81068
		125		65.6863
		0	Control	100
22		31.25	Wax gourd	50.15078
		62.5		28.73273
		125		18.58512
		0	Control	100
23		31.25	Snake gourd	89.79565
		62.5		79.83615
		125		56.90816
		0	Control	100
24		31.25	Taro	39.04361
		62.5		33.0622
		125		18.94942
		0	Control	100
25		31.25	Water spinach	92.99889
		62.5		74.08205

		125		55.60515
		0	Control	100
26		31.25	Amaranth	43.15418
		62.5		29.35198
		125		15.46965
		0	Control	100
27		31.25	Ficus	61.49484
		62.5		35.26241
		125		28.046
		0	Control	100
28		31.25	Green Taro	57.85498
		62.5		26.9059
		125		21.58802
		0	Control	100
29		31.25	Bottle gourd	93.41513
		62.5		68.97771
		125		35.55794

		0	Control	100
30		31.25	Daikon	98.81902
		62.5		79.41137
		125		60.71676
**Fruits**				
		0	Control	100
		31.25	VS	37.26482
Std		62.5		36.69377
		125		16.58829
		0	Control	100
1		31.25	Apple	59.24997
		62.5		31.32724
		125		25.84073
		0	Control	100
2		31.25	Guava	21.37458
		62.5		14.75025
		125		8.889187
		0	Control	100
3		31.25	Malta	58.05018

		62.5		40.34217
		125		57
		0	Control	100
4		31.25	Indian Apple	100.5836
		62.5		90.48336
		125		63.06942
		0	Control	100
5		31.25	Wood Apple	58.04204
		62.5		34.97311
		125		26.88817
		0	Control	100
6		31.25	Pomelo	94.20154
		62.5		78.86229
		125		60.15024
		0	Control	100
7		31.25	Papaya	87.25583
		62.5		77.10019
		125		65.29471
		0	Control	100
8		31.25	Indian Goose Berry	99.24325
		62.5		83.10349

		125		73.89579
		0	Control	100
9		31.25	Carambola	94.74066
		62.5		88.04455
		125		51.98683
		0	Control	100
10		31.25	Banana	66.51741
		62.5		34.89865
		125		22.70365
		0	Control	100
		31.25	Hog Plum	98.40354
11		62.5		86.33898
		125		75.10962
		0	Control	100
12		31.25	Pomegranate	98.5009
		62.5		86.72561
		125		69.7222
		0	Control	100
13		31.25	Indian Jujube	98.60948
		62.5		83.41379
		125		65.34101

				0
		0	Control	100
14		31.25	Lemon	63.19375
		62.5		27.48578
		125		19.96628
				#DIV/0!
		0	Control	100
15		31.25	Lemon peel	19.86442
		62.5		13.23835
		125		6.565735
		0	Control	100
16		31.25	Custard Apple	98.57289
		62.5		83.81555
		125		63.19031
		0	Control	100
17		31.25	Jambolan	98.62758
		62.5		81.148
		125		63.08137

Supplementary Fig. S1

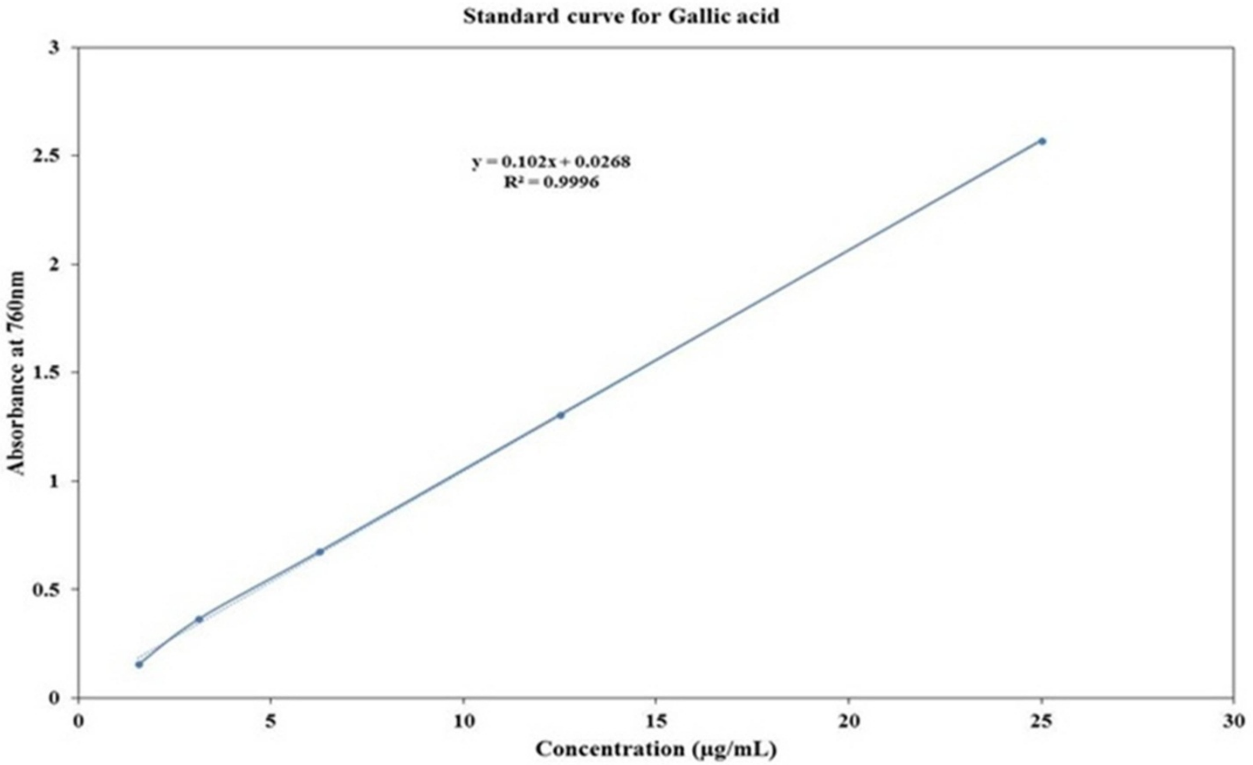

Supplementary Figure S1: Standard curve of gallic acid for the determination of total phenolics.

Supplementary Fig. S2

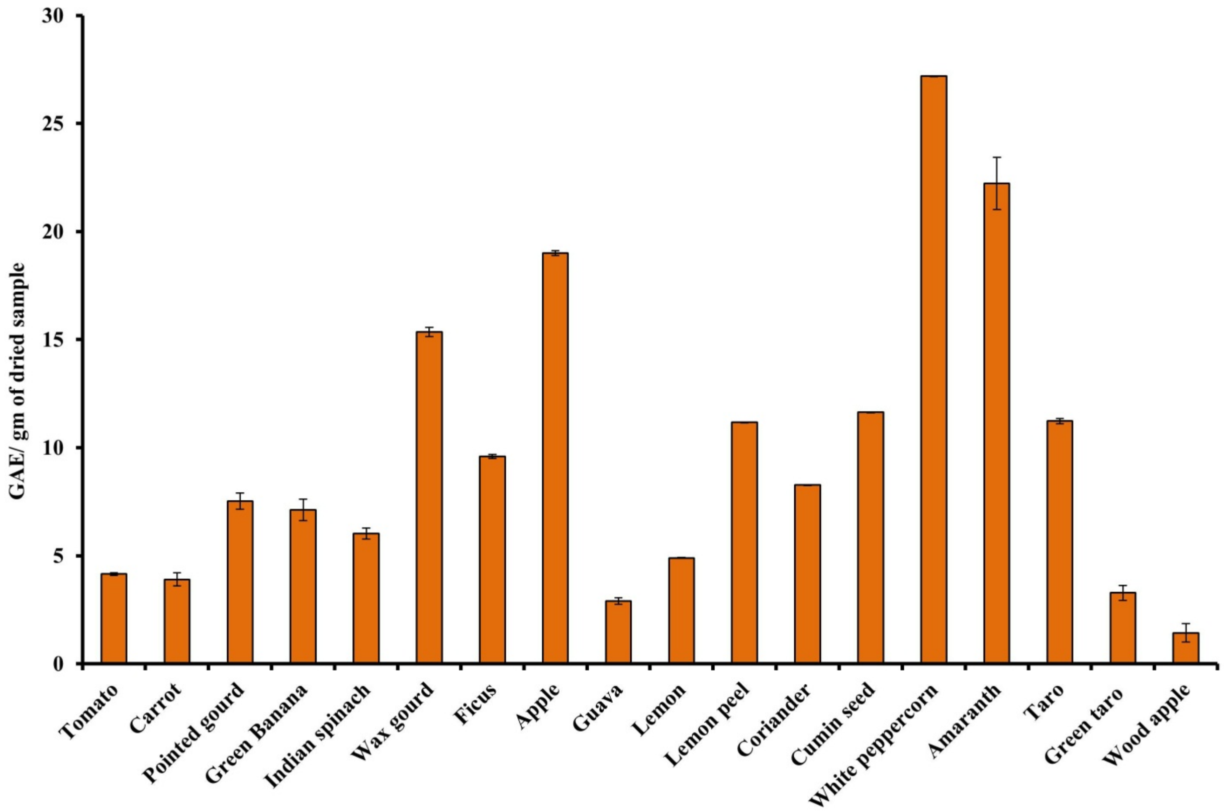

Supplementary Figure S2: Determination of total phenolic contents (mg/gm plant extract in GAE) of vegetables, fruits, and spices. Data are expressed as the mean ± SD (n=3) for all tested doses.

Supplementary Fig. S3

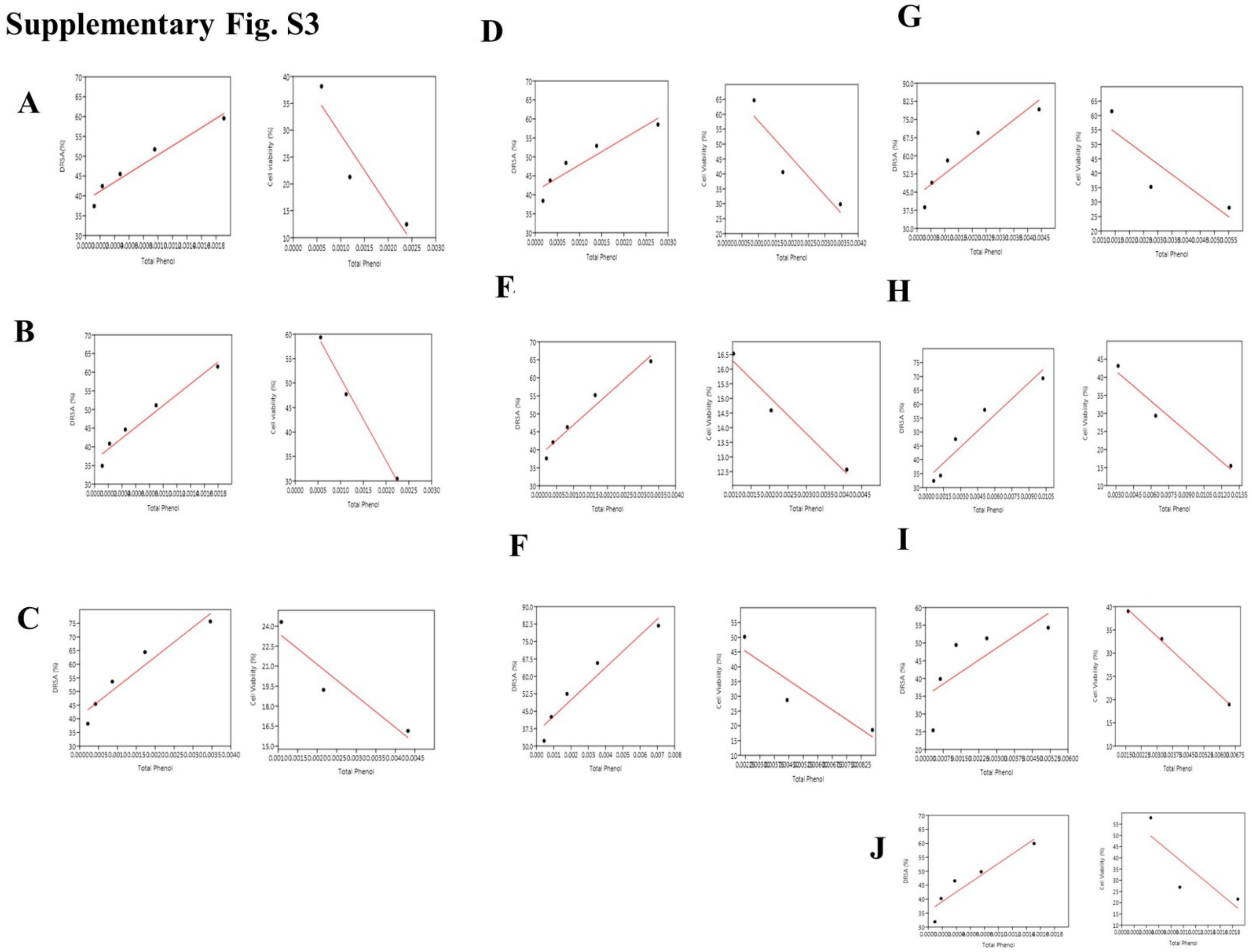

Supplemen[illegible]ell viability of the dietary vegetables. (A) Tomato, (B) Carrot, (C) Pointed gourd, (D) Green banana, (E) Indian spinach, (F) Wax gourd, (G) Ficus, (H) Amaranth, (I) Taro, (J) Green taro. Data are expressed as the mean ± SD (n = 3, *p < 0.05 and **p < 0.001) for all tested doses.

Supplementary Fig. S4

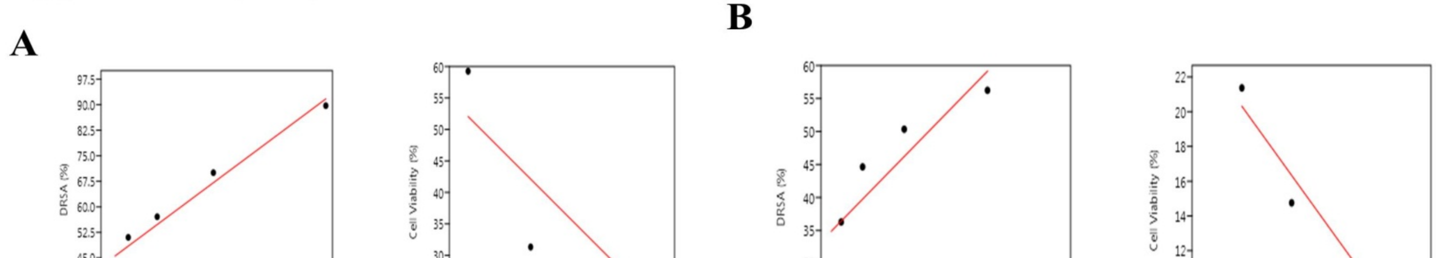

Supplementary Figure S4: Relationship between the total phenolic contents and % of DPPH free radical scavenging and cell viability of the dietary fruits. (A) Apple, (B) Guava, (C) Lemon, (D) Lemon peel, (E) Wood apple. Data are expressed as the mean ± SD (n = 3, *p < 0.05 and **p < 0.001) for all tested doses.

Supplementary Fig. S5

A

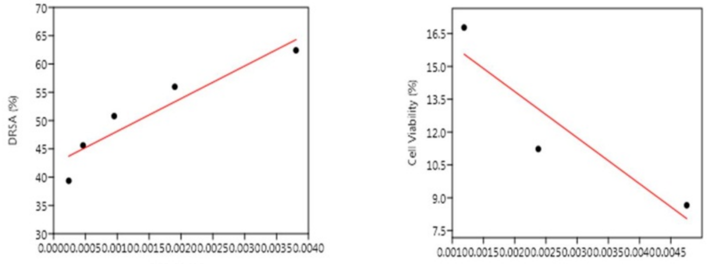

Supplementary Figure S5: Relationship of between the total phenolic contents and % of DPPH free radical scavenging and cell viability of the dietary spices. Where (A) Coriander, (B) Cumin seed, (C) White peppercorn. Data are expressed as the mean ± SD (n = 3, *$p < 0.05$ and **$p < 0.001$) for all tested doses.

Supplementary Figure Legends

Supplementary Figure S1: Determination of total phenolic contents (mg/gm plant extract in GAE) of vegetables, fruits, and spices. Data are expressed as the mean ± SD ($n=3$) for all tested doses.

Supplementary Figure S2: Standard curve of gallic acid for the determination of total phenolics.

Supplementary Figure S3: Relationship between the total phenolic contents and % of DPPH free radical scavenging and cell viability of the dietary vegetables. (A) Tomato, (B) Carrot, (C) Pointed gourd, (D) Green banana, (E) Indian spinach, (F) Wax gourd, (G) Ficus, (H) Amaranth, (I) Taro, (J) Green taro. Data are expressed as the mean ± SD ($n = 3$, *$p < 0.05$ and **$p < 0.001$) for all tested doses.

Supplementary Figure S4: Relationship between the total phenolic contents and % of DPPH free radical scavenging and cell viability of the dietary fruits. (A) Apple, (B) Guava, (C) Lemon, (D) Lemon peel, (E) Wood apple. Data are expressed as the mean ± SD ($n = 3$, *$p < 0.05$ and **$p < 0.001$) for all tested doses.

Supplementary Figure S5: Relationship between the total phenolic contents and % of DPPH free radical scavenging and cell viability of the dietary spices. Where (A) Coriander, (B) Cumin seed, (C) White peppercorn. Data are expressed as the mean ± SD ($n = 3$, *$p < 0.05$ and **$p < 0.001$) for all tested doses.

Supplementary Table Legends

Supplementary Table S1: List of vegetables

Supplementary Table S2: List of fruits

Supplementary Table S3: List of spices

Supplementary Table S4: Determination of anticancer activity using the MTT assay of spices, vegetables and fruits on HeLa cells at concentrations of 31.25 to 125 µg/mL.
